# The Impact of Fascial Manipulation^®^ on Posterior Shoulder Tightness in Asymptomatic Handball Players: A Randomized Controlled Trial

**DOI:** 10.3390/diagnostics14171982

**Published:** 2024-09-07

**Authors:** Anja Barič, Breda Jesenšek Papež, Majda Bastič, Robi Kelc, Peter Brumat, Antonio Stecco

**Affiliations:** 1 Fiziomania, Anja Barič s.p., fizioterapija, 6310 Izola, Slovenia; 2 Institute of Physical and Rehabilitation Medicine, University Medical Centre Maribor, 2000 Maribor, Slovenia; breda.jesensekpapez@ukc-mb.si; 3 Alma Mater Europaea—ECM, 2000 Maribor, Slovenia; majda.bastic@almamater.si; 4 Department of Orthopaedics, University Medical Centre Maribor, 2000 Maribor, Slovenia; robi.kelc@ukc-mb.si; 5 Faculty of Medicine, University of Maribor, 2000 Maribor, Slovenia; 6 Valdoltra Orthopaedic Hospital, 6280 Ankaran, Slovenia; peter.brumat@ob-valdoltra.si; 7 Faculty of Medicine, University of Ljubljana, 1000 Ljubljana, Slovenia; 8 Department of Rehabilitation Medicine, New York University Grossman School of Medicine, New York, NY 10016, USA

**Keywords:** handball, throwing athletes, shoulder, range of motion, posterior shoulder tightness, injury, risk factor, fascia, connective tissue, fascial manipulation

## Abstract

This prospective study aimed to determine the impact of Fascial Manipulation^®^ by Stecco (FM) on the range of motion (ROM) of internal rotation (IR) and horizontal adduction (HADD) in asymptomatic handball players, representing significant risk factors for shoulder injuries. A randomized controlled trial was conducted, with participants randomly assigned to either the investigated group (*N* = 29) receiving a single session of FM or the control group (*N* = 27) receiving no treatment. The ROM for IR and HADD were measured before, immediately after, and one month after the FM session. The investigated group experienced a statistically significant acute increase in glenohumeral IR (14 degrees, *p* < 0.001) and HADD (14 degrees, *p* < 0.001) compared to the control group (*p* < 0.001). The positive effects of FM persisted one month post-treatment, with increased IR ROM by 12 degrees (*p* < 0.001) and HADD ROM by 11 degrees (*p* < 0.001). Participants in the investigated group reported lower subjective tightness/stiffness immediately after (*p* < 0.001) and one month after treatment (*p* = 0.002) compared to the control group. This study demonstrates that a single application of FM effectively improves glenohumeral ROM in the dominant throwing shoulder of asymptomatic handball players. It highlights the immediate and sustained positive effects of FM on IR and HADD. These findings support the use of FM as an effective method for enhancing shoulder ROM and reducing subjective tightness/stiffness. The study was registered at ClinicalTrials.gov (NCT06009367).

## 1. Introduction

Posterior shoulder joint tightness (PST) is defined as a widespread restriction of the posterior soft tissues of the shoulder joint, including contractile and non-contractile elements and bony changes (torsion of the humerus) that are characterized as an adaptation to the loads of the athlete engaged in throwing the ball above shoulder height [1]. Studies relating changes in shoulder range of motion (ROM) and PST focus on loss of internal rotation (IR), with authors also observing reduced horizontal adduction (HADD) [2,3,4,5,6].

An increase in humeral retroversion usually leads to a shift in the entire arc of motion so that increased ER equals decreased IR ROM. However, posterior shoulder soft tissue tightness can result in decreased IR ROM of the joint [7,8,9,10] without a concomitant increase in ER (pathologic GIRD). Pathologic GIRD has been defined as a loss of IR combined with a loss of total rotational motion [11].

In handball, reduced ROM of internal rotation and excessive external rotation have been suggested as risk factors in studies [4,8,9,12,13,14,15]. A reduction in total rotation motion has been associated with shoulder problems in studies [4,15,16,17,18,19]. Large deficits in the range of motion of the dominant shoulder (versus non-dominant) are of particular concern, as the risk of injury appears to increase 2.5- to 9-fold according to studies [15,18,19]. Specifically, glenohumeral (GH) deficits of IR [8] > 20°, reduced total rotation range of motion [20] > 5°, and reduced range of horizontal adduction (HADD) [18] > 16° were all identified as risk factors for throwing-related injuries. Unfortunately, the primary tissue mechanisms responsible for these range of motion deficits remain unclear [2].

Improving PST through targeted interventions could potentially enhance athletic performance and reduce injury risk by addressing these deficits in ROM. By increasing internal rotation and horizontal adduction, athletes may experience more balanced shoulder mechanics, leading to greater efficiency in throwing motions and decreased strain on the shoulder joint. This, in turn, could lower the likelihood of overuse injuries and help maintain long-term shoulder health, which is crucial for sustained athletic performance.

Conservative therapy based on stretch and passive joint and muscular mobilizations can be useful to improve the internal rotation and adduction ROM [21]. Researchers have studied the effects of different stretching techniques to improve HADD and IR ROM [22,23,24]. However, these techniques differentially affected GH joint mobility, introducing additional confusion regarding the choice of optimal stretching techniques [24]. The tension and strain values of muscles, tendons, and fascia show that during passive stretching, the fascia is the first tissue that limits the stretch, suggesting that the fascial tissue is probably the main target of static stretching [25]. Furthermore, no research has examined the efficacy of Fascial Manipulation (FM) according to Stecco (Fascial Manipulation^®^) for the treatment of PST. In the FM the treated tissue is the connective tissue-fascia, so the technique cannot directly influence the bony (humeral retrotorsion) and posterior capsular changes of the GH joint, but we can influence the fascial component of the PST. It has been proposed that the friction caused by manipulation creates localized heat and that, due to the thermosensitive characteristics of fascia, this heat facilitates the transition of hyaluronic acid within the extracellular matrix from a condensed state to a more fluid state, thus restoring the gliding properties of the fascia [26].

The aim of our research was to determine with an experiment whether and how FM affects IR and HADD ROM in handball players. Our hypotheses include an increase in IR and HADD ROM immediately after FM compared to the control group (H1), maintenance of increased ROM one month after FM compared to baseline and the control group (H2), and a decrease in subjective feeling of tightness immediately and one month after FM compared to the control group (H3).

## 2. Materials and Methods

### 2.1. Data Collection

A prospective randomized controlled trial was conducted on handball players from Izola Handball Club (Slovenia) during the first part of the 2022/2023 competition season. The participants were divided into two groups through random allocation. The first group underwent the Fascial Manipulation^®^ (FM) technique by Stecco (referred to as the investigated or experimental group), while the second group served as the control and did not receive FM treatment. Both male and female participants volunteered for the study and belonged to cadet, junior, and senior handball teams representing three different age groups.

The study protocol adhered to the principles outlined in the Declaration of Helsinki and received approval from the National Medical Ethics Committee of the Republic of Slovenia (0120-78/2022/9). The study was registered at ClinicalTrials.gov (NCT06009367). All participants aged 18 years and older provided informed consent to participate in the study, whereas participants under the age of 18 had their parents or guardians sign the informed consent forms on their behalfs.

Demographic information, including age, height (cm), body mass (kg), dominant shoulder side, playing position, years of playing handball, and subjective assessment of tightness in the dominant shoulder joint, was collected from all participants.

### 2.2. Participants

Exclusion criteria for participation in the study included a recent upper extremity injury or surgery that hindered the player’s ability to participate in handball for the past three months, any history of previous shoulder surgery, and the position of goalkeeper. A cluster random sampling strategy was employed to ensure the distribution of participants. The players were randomly assigned to either the investigated group or the control group, with efforts made to achieve a balanced distribution of sex and age within each group. In the investigated group, the FM technique was performed, while the control group did not receive FM treatment.

### 2.3. Testing Procedure and Outcome Measure

All participants underwent three test sessions, including a pre-test, immediate post-test, and one-month post-test. The immediate post-test measurements were taken immediately after the application of FM in the investigated group and after a one-minute waiting period in the control group. The testing was repeated approximately one month later, ranging from 27 to 34 days after the FM intervention. The measurement procedures for all three sessions were standardized and performed in the same manner. Two independent examiners, who were experienced physical therapists, conducted the measurements. The FM technique was administered exclusively by the principal investigator, an experienced physical therapist who did not participate in the measurements. Prior to measuring IR and HADD ROM, participants engaged in a team-specific standard warm-up routine, which included gentle jogging and static and dynamic full-body stretching. However, the warm-up protocol was not standardized for this particular study.

ROM measurements for IR and HADD were recorded using a classic goniometer. For HADD assessment, participants were positioned supine on a standard examination table with both shoulders aligned. The examiner stood on top of the table, facing the participant’s head, and stabilized the lateral edge of the scapula. The participant’s upper limb was positioned at 90 degrees of GH abduction with 90 degrees of elbow flexion. The examiner held the subject’s arm distal to the elbow and passively moved it into HADD. At the first point of resistance, a second examiner used a digital inclinometer to record the amount of motion in degrees (°) by aligning it with the humerus.

Passive IR measurements were taken with the participant lying supine on an examination table, with the shoulder at 90 degrees of abduction in the frontal plane and the elbow at 90 degrees of flexion. The examiner applied a posterior stabilizing force to the acromial process of the scapula and internally rotated the upper limb until the first point of resistance. A second investigator then recorded the amount of movement in degrees by aligning the digital inclinometer with the axis of the forearm.

### 2.4. Intervention

One session of Fascial Manipulation^®^ by Stecco was chosen as the therapeutic procedure for the investigated group. The principal investigator who performed the FM is a qualified therapist with extensive knowledge in manual therapy and holds certificates in FM Levels I and II. The therapist utilized the Stecco model to interpret the dysfunction of the fascial system based on comprehensive information collected, including the participant’s complete medical history, traumatic events, fractures, musculoskeletal dysfunctions, scars, surgical procedures, specific range of motion, and palpation examinations. Using this information, the therapist selected an individualized combination of condensed treatment points, known as centers of coordination (CC) and/or centers of fusion (CF). All CC and/or CF points treated in each individual subject were recorded for documentation purposes.

### 2.5. Data Analysis

The minimal sample size for this study was calculated based on the study by Reed et al. [27]. A power of 90% (1-β), a standard deviation (SD) of 9, and a significance level of 0.05 were used. Cohen’s coefficient (increased clinical efficiency) of d = 10 was chosen. Statistical analyses were conducted using SPSS 25 (IBM SPSS Statistics, Armonk, NY, USA).

The decision to use parametric or non-parametric tests was based on the results of normality tests conducted on the variables beforehand. We evaluated the distribution of our data using the Shapiro-Wilk test. If the results indicated that the data followed a normal distribution, we employed parametric tests, which assume normality. However, if the data significantly deviated from a normal distribution, we opted for non-parametric tests.

To analyse the characteristics of the difference in IR ROM immediately after FM in the investigated group, a parametric *t*-test for two dependent samples was employed. The non-parametric Wilcoxon signed-rank test was used to examine the difference in HADD ROM immediately after FM in the investigated group. The non-parametric Wilcoxon signed-rank test was also utilized to analyse the difference characteristics of IR and HADD immediately after FM in the control group. Furthermore, the non-parametric Mann-Whitney test for two independent samples was applied to assess the difference between the differences of IR and HADD ROM before and immediately after FM between the control and investigated groups.

To examine the changes before FM and after one month following FM in the values of IR and HADD ROM variables in the investigated group, a parametric *t*-test for two dependent samples was employed. For the control group, a parametric *t*-test for two dependent samples was used to analyse the changes before FM and after one month following FM in the values of IR variables, while the non-parametric Wilcoxon signed-rank test was used for HADD variables. To test the difference between the mean values of the variable (change in IR one month after FM) between the groups, a parametric *t*-test for two independent samples was employed. Additionally, the non-parametric Mann-Whitney test was used to examine the characteristics of the change in HADD one month after FM between the groups.

To test H3, the variable used was the subjective assessment of the feeling of tightness/stiffness, which was measured on a 7-point interval scale (1=I do not feel any tightness, 7=I feel very tight/I have a feeling of stiffness in the shoulder joint). The non-parametric Wilcoxon signed-rank test was utilized to determine the significance of the difference in the variable within each group immediately after the test and one month after the test. The non-parametric Mann-Whitney test was used to analyse the characteristics of the difference in the subjective evaluation of the feeling of tightness/spasm between the two groups.

## 3. Results

In our research, male participants (*N* = 2) and female participants (*N* = 1) who had undergone shoulder joint surgery in the past were excluded. Additionally, 14 goalkeepers were excluded from the target group as they do not experience the same level of strain on their upper limbs during the game. Three handball players declined to participate in the study, resulting in a final sample size of 56 participants (27 females and 29 males). Among them, 27 participants (14 females and 13 males) were assigned to the control group, while 29 participants (15 females and 14 males) were assigned to the experimental group. Descriptive statistics for the participants in the research can be found in Table 1.

Supplementary Table S1 provides a list of individual centers for coordination (CC) and centers of fusion (CF) that were considered for each subject in the investigated group during the FM procedure (Supplementary Table S1).

### 3.1. The Immediate Effect of the Fascial Manipulation on Shoulder Internal Rotation (IR) and Horizontal Adduction (HADD) Range of Motion (ROM)

The first hypothesis aimed to examine the immediate effect of FM on shoulder IR and HADD ROM and compare it with the control group. The results revealed a statistically significant increase in IR by 13.97° (95% CI, 11.258 to 16.673, t(28) = 10.565, *p* < 0.001) and HADD by 14.49° (*p* < 0.001) immediately after performing FM in the investigated group. In contrast, no significant changes were observed in the control group, where IR before FM was 42.78° and after one minute of rest was 42.96° (*p* = 0.317), and HADD before FM was 40.74° and after one minute of rest was 40.19° (*p* = 0.083). The investigated group showed an increase in IR from 36.03 ± 7.49° to 50.00 ± 7.56° and an increase in HADD from 35.00° to 50.00° immediately after performing FM.

The median of the differences in IR and HADD ROM in the investigated group (15.0°) was significantly higher than in the control group (0.00°), where no changes were observed (*p* < 0.001). These findings support our research hypothesis, which posited that IR and HADD ROM would increase after performing FM in the investigated group compared to the control group.

### 3.2. One-Month Effect of Fascial Manipulation on Shoulder Internal Rotation (IR) and Horizontal Adduction (HADD) Range of Motion (ROM)

Upon evaluating the changes in IR and HADD ROM variables before and one month after performing FM, we observed that in the investigated group, the increase in IR and HADD ROM was sustained even after one month compared to the pre-FM state. Specifically, one month after FM, the IR ROM in the investigated group increased from 36.03 ± 7.49° to 48.45 ± 5.02°, with a significant increase of 12.41° (95% CI, 9.466 to 15.261, t(28) = 8.628, *p* < 0.001). Similarly, the HADD ROM increased from 35.69 ± 8.21° to 47.07 ± 6.75° in the investigated group, with a significant increase of 11.38° (95% CI, 8.600 to 14.159, t(28) = 8.387, *p* < 0.001).

On the other hand, in the control group, there were no statistically significant changes in the median values of IR and HADD ROM one month after FM compared to the pre-FM state. Although IR increased from 42.78 ± 8.59° to 46.11 ± 7.12° in the control group, the increase was not statistically significant at 3.33° (95% CI, −0.306 to 6.972, t(26) = 1.883, *p* = 0.071). Similarly, the median value of HADD did not change in the control group (Me = 45° before and Me = 45° one month after, *p* = 0.123).

The average difference in IR between before FM and one month after FM was 3.33 ± 9.2° in the control group and 12.41 ± 7.75° in the investigated group. The difference between these mean values was 9.08° and statistically significant (t(54) = −4.005, *p* < 0.001). The median changes in HADD one month after FM were 0° in the control group and 10.00° in the investigated group, with a significant difference between the two medians (*p* < 0.001).

These findings confirm our research hypothesis that the increase in IR and HADD ROM is maintained even one month after FM in the investigated group, in contrast to the control group where no significant changes in IR and HADD ROM were observed.

**Figure 1:**
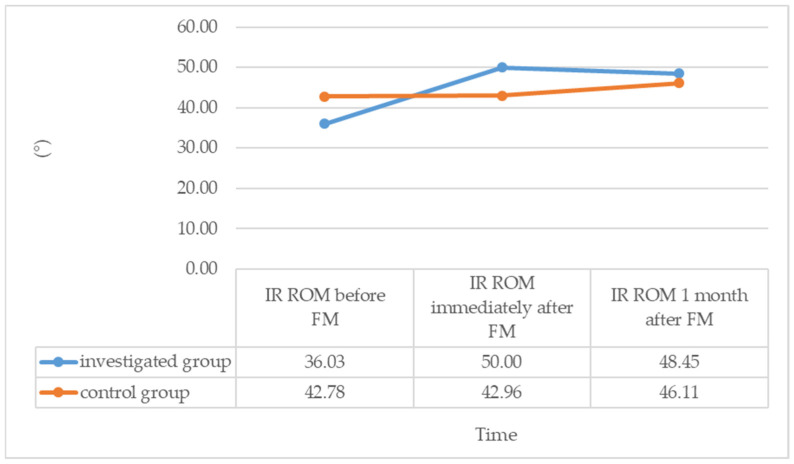
Average values of the shoulder IR ROM during the investigation period for both groups. Shoulder internal rotation (IR) range of motion (ROM). Fascial Manipulation^®^ by Stecco (FM).

**Figure 2:**
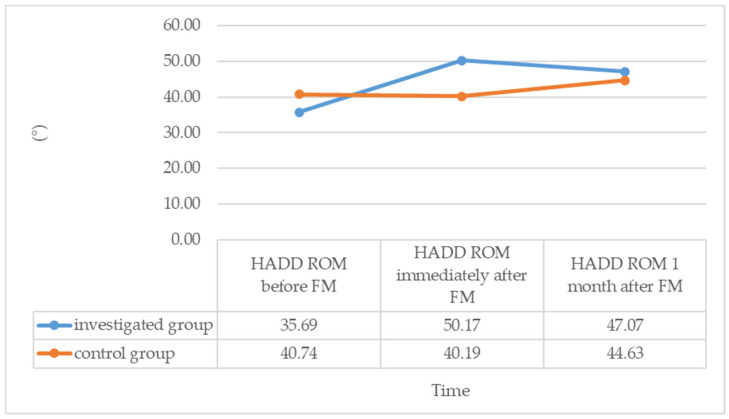
Average values of the shoulder HADD ROM during the investigation period for both groups. Shoulder horizontal adduction (HADD) range of motion (ROM). Fascial Manipulation^®^ by Stecco (FM).

Figure 1 and Figure 2 illustrate the changes in IR and HADD ROM during the study period in both the investigated and control groups.

In Table 2, Table 3, Table 4 and Table 5, the changes in IR ROM and HADD ROM during the study period for the investigated group and control group are presented.

### 3.3. Assessing Changes in Subjective Feeling of Tightness: Immediate and One-Month Effects of Fascial Manipulation in Each Group and Group Comparisons

The results indicate that FM has a positive effect on reducing the feeling of tightness. Immediately after FM in the investigated group, there was a significant reduction in the subjective assessment of tightness (*p* < 0.001), and this reduction was maintained even one month after the FM implementation. In the control group, there was no immediate change in the feeling of tightness (*p* = 0.317), but over the course of one month, there was a significant decrease (*p* < 0.001).

The average change in subjective tightness scores between before and after FM was −1.103 in the investigated group and −0.04 in the control group. The medians in both groups were 0. The median differences between subjective ratings were statistically significant (*p* < 0.001), indicating a difference in subjective tightness scores immediately after FM between the groups.

Similarly, the average change in subjective tightness scores between before FM and one month after FM was −1.83 in the investigated group and −0.85 in the control group. The medians in both groups were −1. The median differences between subjective ratings were statistically significant (*p* = 0.002), indicating a difference in subjective tightness scores one month after FM between the groups.

These findings suggest that FM has a positive and sustained effect on reducing the subjective sensation of tightness, with greater improvements observed in the investigated group compared to the control group.

## 4. Discussion

Due to the extreme range of motion (ROM) and high arm velocities that occur during throwing, the glenohumeral (GH) joint is subjected to enormous amounts of forces [15]. Repetitive movements with very high loads usually cause specific anatomical adaptations on the body, especially on the soft tissue and bone structure of the dominant GH joint [7,8,9,28,29,30,31]. A decrease in IR and HADD ROM are indicators of PST.

Our study aimed to investigate whether targeting the fascial component of PST with Fascial Manipulation^®^ (FM) could effectively reduce PST. The deep fascia is a continuous layer that extends from the trunk to the upper and lower limbs, playing a crucial role in transmitting loads parallel to the joints. It also serves as the attachment and insertion site for approximately 30% of muscle fibers [32]. The presence of constant non-physiological tension in the fascial segment can lead to the development of adaptive fibers and subsequent pain both distally and proximally [33]. Based on this understanding, various musculoskeletal dysfunctions, including chronic shoulder pain [34,35], adhesive capsulitis [36], TMJ disorders [33], and chronic low back pain [37], have been effectively treated using FM. In line with this theory, we sought to explore the potential benefits of FM in addressing PST.

We examined the immediate effect of FM on shoulder joint range of motion (ROM) in internal rotation (IR) and horizontal adduction (HADD) and compared it to the control group. In the investigated group, we observed a statistically significant increase in IR and HADD immediately after the FM treatment, compared to the baseline measurement and the control group, where no changes were observed. Our findings are consistent with studies that employed manual techniques in a single treatment session and measured ROM before and immediately after the intervention. Similar immediate and short-term improvements in IR and HADD have been reported in studies investigating the effects of instrumented soft-tissue mobilization (IASTM) [38,39,40], kinesiology tape [41], muscle energy techniques (MET) [27,42], dry needling of myofascial trigger points [43,44], and joint mobilization [45]. These findings suggest that various manual interventions can produce immediate improvements in shoulder ROM, similar to the effects observed with FM in our study. However, there is currently a lack of direct comparative studies between FM and other treatment methods. This gap in the literature underscores the need for future research to directly compare FM with other established therapies. Such comparative studies would offer a more comprehensive understanding of the relative efficacy, benefits, and limitations of FM in treating musculoskeletal conditions, thus informing clinical practice and guiding treatment decisions more effectively.

During the overhead throw and serve, the shoulder is highly stressed due to the need for an extreme eccentric contraction of the external rotators during the deceleration phase [46]. PST in a certain population can hypothetically develop due to the protective reflex activity of the infraspinatus, teres minor, or posterior deltoid in response to an afferent stimulus from the GH joint capsule [47,48,49,50]. CC and CF points that were affected the most in our cases were right in that myofascial area: RE-ME-HU (teres major, posterior fibers of deltoideus, latissimus dorsi, long head triceps muscle), ER-HU (distal part of the infraspinatus-insertion muscle), and RE-LA-HU (infraspinatus, teres minor, posterior fibers of deltoid muscle). We hypothesize that the repetitive overhead throwing motion over time may result in changes not only in the shoulder’s bony, capsular, and muscle/tendon structures but also in the fascial tissue. When employing FM, it is important to consider additional mechanisms that can impact joint movement and function. For instance, FM has the potential to release the fascia in the posterior-inferior region of the shoulder, thereby influencing GH joint translation, rectifying the motor plan, and enhancing joint arthrokinematics. This approach aligns with the strategy recommended by Wilk et al. [51] to indirectly enhance ROM in IR through myofascial-directed intervention. Through a review of the literature, Carla Stecco [25] supports the role of non-muscular structures such as fascia in flexibility, stretch sensing, and limited maximal ROM. The increased viscosity of the deep fascia, due to the increased viscosity of hyaluronic acid (HA) molecules, prevents normal sliding of the fascia during movement and inhibits normal proprioception and muscle function [35]. Just as we proved the success of FM in improving shoulder joint ROM, other authors also report successful, immediate improvement in joint mobility using this method [52,53,54,55]. There are several theories that explain the pathophysiological and pathomechanical processes that follow injury or overuse of myofascial tissue, ranging from the cellular level (viscoelasticity, piezoelectricity, tensegrity, etc.) to the global level (force transmission, sliding, fluid dynamics, hysteresis, innervation, sensitization, etc.) [56]. With FM, we were able to influence all these processes and thereby improve the mobility of the shoulder joint. The manual technique itself is based on raising local heat by friction using the elbow, knuckle, or fingertip on coordination centers (CC) and centers of fusion (CF) [57]. Due to its mechanical and thermal effect, FM changes the viscosity of HA in the fascia, which represents the thixotropic effect. Thixotropic is the property of a substance to reduce its viscosity when it is shaken or stirred and then to solidify again when left to stand. HA (glucosaminoglycan) within the fascia, which provides gliding, hydration, absorbs compressive force, and improves fascial movement, is highly dependent on its viscous state [58]. With the therapy, we were able to influence the basic substance of the deep fascia in the condensed points and thereby increase the sliding between the layers of the fascia [34]. The newly acquired relaxation of the connective tissue after FM can spread along the entire myofascial sequence, diagonally or spirally, thereby restoring physiological balance [34]. Hence, in addition to targeting conventional points around the shoulder, such as the teres major and minor, infraspinatus, and posterior deltoid muscle, and based on the physical examination and FM guidelines, several points were also selected on the torso and contralateral limb.

In the second hypothesis, we investigated the effect of FM on IR and HADD ROM one month after the completion of FM treatment. The results demonstrated that in the group undergoing investigation, there was a sustained increase in IR and HADD ROM even one month after the FM treatment, compared to the pre-treatment state. Conversely, no changes in IR and HADD ROM were observed in the control group. When comparing the measurements immediately after the FM treatment (as mentioned in the first hypothesis), a slight reduction in IR of 1.554° (from 13.966° to 12.412°) and in HADD of 3.101° (from 14.48° to 11.379°) was noted one month later. These findings suggest that the positive effects on shoulder joint mobility following a single session of FM can still be experienced for up to one month, nearly to the same extent as immediately after the treatment. It is important to approach these results with caution, as the persistence of effects from a single session is relatively exceptional and requires further investigation to verify long-term outcomes. Additional research is needed to confirm these findings and understand the mechanisms behind the observed effects. Studies by Ćosić et al. [59], Rajasekar and Marchand [60], and Brandolini et al. [52] provide some support for the long-term effectiveness of FM, but further verification is necessary to substantiate these preliminary observations.

One of the objectives of our research was to assess the subjective perception of tightness among the participants before FM, immediately after FM, and one month after FM treatment, and compare it with the control group. Based on the participants’ subjective evaluations, it can be concluded that the sensation of tightness decreases immediately after FM and continues to persist even one month after the treatment. The feeling of tightness was reported to be less compared to the control group. It is important to note that these findings are based on subjective assessments, which are considered less reliable than objective measures. However, they provide valuable insights into the participants’ opinions and perceptions.

### Limitations

This study is subject to certain limitations. Firstly, the sample size was calculated to ensure representativeness, but the inclusion of handball players from different age categories may have led to some heterogeneity within the sample.

Another limitation is that we did not gather information from the subjects regarding the intensity and frequency of their training and matches during the study period. This lack of data could potentially have influenced our results. Also, the lack of standardization in the warm-up protocol might have affected the ROM measurements and contributed to variability in our results. Furthermore, the methodology employed in this study was unable to differentiate whether reduced IR mobility in the shoulder joint was primarily caused by capsular stiffness, humeral retroversion, posterior soft-structure stiffness, or a combination of these factors. To determine the underlying causes of reduced IR mobility, additional diagnostic tests would be necessary, but they were beyond the scope of our research.

In terms of the limitations specific to the implementation of FM, the main drawback is the lack of objective measurements for the strength, direction, speed, and depth of the manual technique. These characteristics are solely dependent on the skills and judgment of the investigator, as well as the selection of treatment points and the duration of therapy.

## 5. Conclusions

Posterior shoulder tightness (PST) is recognized as an adaptation seen in overhead athletes due to repetitive motions and excessive loads on the shoulder. It is also considered as a potential predictor of shoulder injuries. Our study demonstrates that Stecco’s fascial treatment (Fascial Manipulation^®^) appears effective in restoring range of motion in internal rotation (IR) and horizontal adduction (HADD), which are commonly restricted in individuals with PST. Notably, the positive effects of Fascial Manipulation^®^ were observed to persist for up to one month following therapy, suggesting potential long-term efficacy.

Based on our findings, we propose that restricted or altered fascial tissue may contribute to the development of PST; however, this proposed link warrants further investigation to fully understand its role and implications. In the context of handball, incorporating manual treatment targeting the fascial structures, particularly the posterior aspect of the shoulder joint, may be beneficial. Furthermore, considering the role of the kinetic chain during treatment is essential for optimizing outcomes.

These recommendations may offer valuable insights for managing PST in handball players and underscore the importance of exploring fascial tissue as a potential underlying mechanism of this condition. Future research should aim to validate these findings and further elucidate the effectiveness of Fascial Manipulation^®^ compared to other therapeutic options.

## Figures and Tables

**Table 1:** Descriptive statistics by sex.

		Female	Male	Total
*N*		27 (48.2%)	29 (51.8%)	56 (100%)
Mean Age (years)		18.93 ± 3.02	20.07 ± 5.20	19.48 ± 4.21
Mean Height (cm)		169.83 ± 6.53	183.59 ± 8.75	176.46 ± 10.30
Mean Weight (kg)		65.31 ± 9.64	79.74 ± 15.58	72.27 ± 14.66
Mean Years of playing		10.62 ± 3.88	10.81 ± 4.32	10.71 ± 4.06
Throwing arm	Left	2 (3.6%)	5 (8.9%)	7 (12.5%)
	Right	27 (48.2%)	22 (39.3%)	49 (87.5%)
Playing position	Left wing	5 (8.9%)	5 (8.9%)	10 (17.9%)
	Right wing	4 (7.1%)	2 (3.6%)	6 (10.7%)
	Left back	7 (12.5%)	6 (10.7%)	13 (23.2%)
	Right back	1 (1.8%)	3 (5.4%)	4 (7.1%)
	Middle back	5 (8.9%)	6 (10.7%)	11 (19.6%)
	Pivot	7 (12.5%)	5 (8.9%)	12 (21.4%)
Investigated group		15 (26.8%)	14 (25.0%)	29 (51.8%)
Control group		14 (25.0%)	13 (23.2%)	27 (48.2%)

**Table 2:** The changes in IR ROM immediately after FM for the investigated group and control group.

	Variable	Mean	Standard Deviation	Median	Min	Max	***p***
Investigated group	IR ROM before FM (°)	36.03	7.49	35.00	20	50	<0.001
	IR ROM immediately after FM (°)	50.00	7.56	50.00	35	70	
	Difference IR ROM immediately after FM (°)	13.97	7.12	15.00	0.00	30.00	
Control group	IR ROM before FM (°)	42.78	8.59	40.00	20.00	60.00	0.317
	IR ROM immediately after FM (°)	42.96	8.35	40.00	20.00	60.00	
	Difference IR ROM immediately after FM (°)	0.185	0.96	0.00	0.00	5.00	

Shoulder internal rotation (IR) range of motion (ROM). Fascial Manipulation^®^ by Stecco (FM).

**Table 3:** The changes in HADD ROM immediately after FM for the investigated group and control group.

	Variable	Mean	Standard Deviation	Median	Min	Max	***p***
Investigated group	HADD ROM before FM (°)	35.69	8.21	35.00	20	50	<0.001
	HADD ROM immediately after FM (°)	50.17	8.71	50.00	30	60	
	Difference HADD ROM immediately after FM (°)	14.49	5.40	15.00	5.00	25.00	
Control group	HADD ROM before FM (°)	40.74	12.76	45.00	5.00	60.00	0.083
	HADD ROM immediately after FM (°)	40.19	13.26	45.00	5.00	60.00	
	Difference HADD ROM immediately after FM (°)	−0.556	1.601	0.00	−5.00	0.00	

Shoulder horizontal adduction (HADD) range of motion (ROM). Fascial Manipulation^®^ by Stecco (FM).

**Table 4:** The changes in IR ROM one month after FM for the investigated group and control group.

	Variable	Mean	Standard Deviation	Median	Min	Max	***p***
Investigated group	IR ROM before FM (°)	36.03	7.49	35.00	20	50	<0.001
	IR ROM 1 month after FM (°)	48.45	5.02	50.00	40	60	
	Difference IR ROM 1 month after FM (°)	12.41	7.75	10.00	0.00	30.00	
Control group	IR ROM before FM (°)	42.78	8.59	40.00	20.00	60.00	0.071
	IR ROM 1 month after FM (°)	46.11	7.12	45.00	30	65	
	Difference IR ROM 1 month after FM (°)	3.33	9.20	0.00	−15.00	20.00	

Shoulder internal rotation (IR) range of motion (ROM). Fascial Manipulation^®^ by Stecco (FM).

**Table 5:** The changes in HADD ROM one month after FM for the investigated group and control group.

	Variable	Mean	Standard Deviation	Median	Min	Max	***p***
Investigated group	HADD ROM before FM (°)	35.69	8.21	35.00	20	50	<0.001
	HADD ROM 1 month after FM (°)	47.07	6.75	45.00	30	60	
	Difference HADD ROM 1 month after FM (°)	11.38	7.31	10.00	0.00	30.00	
Control group	HADD ROM before FM	40.74	12.76	45.00	5.00	60.00	0.123
	HADD ROM 1 month after FM (°)	44.63	6.92	45.00	25	60	
	Difference HADD ROM 1 month after FM (°)	3.90	11.55	0.00	−15.00	40.00	

Shoulder horizontal adduction (HADD) range of motion (ROM). Fascial Manipulation^®^ by Stecco (FM).

## Data Availability

Derived anonymized data supporting findings of this study are available from the corresponding author upon request.

## Supplementary Materials

The following supporting information can be downloaded at: https://www.mdpi.com/article/10.3390/diagnostics14171982/s1, Supplementary Table S1: The individual centers of coordination (CC) and centers of fusion (CF) that were considered for each subject in the investigated group during Fascial Manipulation (FM).
