# Supplementary material for: The Impact of Fascial Manipulation® on Posterior Shoulder Tightness in Asymptomatic Handball Players: A Randomized Controlled Trial
Source: Diagnostics (Basel). 2024 Sep 7;14(17):1982. doi: 10.3390/diagnostics14171982 (PMC11394302; doi:10.3390/diagnostics14171982)
Supplement: Supplementary file 1 [file diagnostics-14-01982-s001.zip › diagnostics-3152268-supplementary.pdf]

Table S1: The individual centers of coordination (CC) and centers of fusion (CF) that were considered for each subject in the investigated group during Fascial Manipulation (FM).

Subject #	1st treated point	2nd treated point	3rd treated point	4th treated point	5th treated point	6th treated point
**1**	ER-CL rt	RE-LA-TH rt	RE-ME-HU rt	ER-SC rt	RE-ME-SC2 rt	IR-SC rt
**2**	RE-ME-HU rt	RE-LA-HU rt	ER-HU rt	AN-HU rt	AN-DI rt	
**3**	AN-HU rt	RE-CX lt	RE-GE lt	RE-ME-HU rt	RE-LA-HU rt	RE-LA-SC1 rt
**4**	AN-LA-HU rt	RE-LA-CX lt	RE-LA-TA lt	RE-ME-HU rt	RE-LA-HU rt	AN-LA-CA1 rt
**5**	RE-LA-TA lt	RE-LA-CX lt	RE-GE lt	RE-ME-HU rt	RE-LA-HU rt	AN-LA-CA1 rt
**6**	AN-HU rt	RE-CX lt	RE-GE lt	RE-ME-HU rt	RE-LA-HU rt	RE-ME-SC2 rt
**7**	RE-ME-HU lt	RE-LA-HU lt	ER-HU lt	RE-LA-CX lt	IR-SC lt	IR-HU lt
**8**	RE-ME-HU rt	RE-LA-HU	ER-HU rt	RE-ME-CX lt	RE-ME-SC2 rt	
**9**	RE-ME-HU rt	RE-LA-HU rt	ER-HU-lt	RE-ME-CX lt	RE-ME-SC2 rt	
**10**	RE-ME-HU lt	RE-LA-HU lt	ER-HU lt	RE-ME-CX lt	R-ME-SC2 lt	
**11**	RE-ME-HU rt	RE-LA-HU rt	ER-HU rt	RE-ME-CX lt	RE-ME-SC2 rt	
**12**	RE-LA-CX lt	RE-SC rt	RE-HU rt	RE-LA-HU rt	IR-HU rt	AN-LA-HU rt
**13**	RE-LA-CX lt	RE-SC rt	RE-HU rt	RE-LA-HU rt	IR-HU rt	AN-LA-HU rt
**14**	RE-LA-CX lt	RE-LA-TA lt	RE-HU rt	ER-SC rt	RE-LA-HU rt	RE-ME-HU rt
**15**	ER-CX lt	RE-SC rt	ER-HU rt	ER-SC rt	RE-LA-HU rt	RE-ME-HU rt
**16**	ER-CX lt	RE-SC rt	RE-HU rt	ER-SC rt	RE-LA-HU rt	RE-ME-HU rt
**17**	RE-LA-CX lt	RE-LA-GE lt	RE-HU rt	ER-SC rt	RE-LA-HU rt	
**18**	ER-HU lt	RE-LA-GE lt	ER-CX lt	ER-SC rt	RE-LA-HU rt	RE-ME-HU rt
**19**	RE-LA-CX lt	RE-SC rt	RE-HU rt	ER-SC rt	RE-LA-HU rt	
**20**	RE-LA-CX lt	RE-SC rt	RE-HU rt	ER-SC rt	RE-LA-HU rt	
**21**	RE-LA-CX lt	AN-HU rt	RE-HU rt	ER-SC rt	RE-LA-HU rt	RE-ME-HU rt

**22**	RE-LA-CX lt	RE-SC rt	RE-HU rt	ER-HU rt	RE-LA-HU rt	RE-ME-HU rt
**23**	RE-LA-CX lt	RE-LA-CU rt	RE-HU r	ER-HU rt	RE-LA-HU rt	RE-ME-HU rt
**24**	RE-LA-CX lt	RE-SC rt	ER-HU rt	ER-SC rt	RE-LA-HU rt	
**25**	RE-LA-CX lt	RE-LA-TA lt	ER-HU rt	ER-SC rt	RE-LA-HU rt	RE-ME-HU rt
**26**	RE-ME-CX lt	ER-HU rt	RE-HU rt	ER-SC rt	RE-LA-HU rt	
**27**	ER-CX lt	ER-HU rt	RE-HU rt	ER-SC rt	RE-LA-HU rt	
**28**	ER-CX lt	ER-HU rt	RE-HU rt	ER-SC rt	RE-LA-HU rt	
**29**	RE-LA-CX lt	ER-HU rt	RE-HU rt	ER-SC rt	RE-LA-HU rt	
